# Adult ALCAPA: from histological picture to clinical features

**DOI:** 10.1186/s13019-020-1048-y

**Published:** 2020-01-13

**Authors:** Hiroshi Kubota, Hidehito Endo, Hikaru Ishii, Hiroshi Tsuchiya, Yusuke Inaba, Katsunari Terakawa, Yu Takahashi, Mio Noma, Kazuya Takemoto, Seiichi Taniai, Konomi Sakata, Kyoko Soejima, Hiroaki Shimoyamada, Hiroshi Kamma, Hayato Kawakami, Yukihiro Kaneko, Satoru Hirono, Daisuke Izumi, Kazuyuki Ozaki, Tohru Minamino, Hideaki Yoshino, Kenichi Sudo

**Affiliations:** 1 Department of Cardiovascular Surgery, Kyorin University, 6-20-2, Shinkawa, Mitaka, Tokyo, 181-8611 Japan; 2 Department of Cardiac Surgery, University of Tokyo, Tokyo, Japan; 3 Department of Cardiovascular Surgery, National Disaster Medical Center, Tachikawa, Japan; 4 Department of Cardiovascular Surgery, Tokyo Metropolitan Children’s Medical Center, Fuchu, Japan; 5 Department of Internal Medicine, Jyukoukai Hospital, Miyoshi, Japan; 6 Department of Cardiology, Kyorin University, Mitaka, Japan; 7 Department of Pathology, Kyorin University, Mitaka, Japan; 8 Department of Anatomy, Kyorin University, Mitaka, Japan; 9 Department of Cardiovascular Surgery, National Center for Child Health and Development, Tokyo, Japan; 10 Department of Cardiovascular Biology and Medicine, Niigata University Graduate School of Medical and Dental Sciences, Niigata, Japan; 11 Jiseikai Nomura Hospital, Mitaka, Japan

**Keywords:** Congenital heart disease, ALCAPA, BWG syndrome, Slow flow phenomenon, Sudden death, Coronary angiography, ICD implantation, Ventricular arteriole, Ventricular fibrillation, Cardiac pathology

## Abstract

**Background:**

Anomalous left coronary artery from the pulmonary artery (ALCAPA) is a rare congenital coronary anomaly that results in high mortality if left untreated. Our aim was to extend our knowledge of the histological, angiographic, and clinical characteristics of ALCAPA in order to deepen our understanding of this rare entity.

**Case presentation:**

We were involved in the assessment, treatment, and pathological evaluation of two adult ALCAPA patients who were rescued from ventricular fibrillation and then surgically treated to establish a dual coronary artery system. Histological studies indicated various chronic ischemic changes in the myocardium, patchy fibrosis**,** and severely thickened arteriolar walls in both ventricles. The first patient is alive and well 11.5 years after surgical correction without any implantable cardioverter defibrillator (ICD) activations. The second patient required re-do surgery 9 months after the initial operation but subsequently died. Histologically, chronic ischemic alteration of the myocardium and thickened arteriolar walls persisted even after surgical correction, and coronary angiography (CAG) showed an extremely slow flow phenomenon even after surgical correction in both patients. The average postoperative opacification rate in the first case was 7.36 + 1.12 (*n* = 2) in the RCA, 3.81 + 0.51 (*n* = 3) in the left anterior descending (LAD) artery, and 4.08 + 0.27 (*n* = 4) in the left circumflex (LCx) artery. The slow flow phenomenon may represent persistent high arteriolar resistance in both ventricles.

**Conclusions:**

Seldom reported or new findings in adult ALCAPA were identified in two cases. More frequent diagnosis of adult ALCAPA can be expected because of the widespread availability of resuscitation and more advanced diagnostic modalities. Accumulation of pathological and clinical findings and confirmation of the long-term follow-up results after treatment may contribute to expanding our knowledge of this rare entity and establishing optimal treatment.

## Background

Congenital abnormalities of the coronary arteries are present in approximately 0.3–0.8% of the population [1, 2)].

Anomalous origin of the left coronary artery from the pulmonary artery (ALCAPA) is an extremely rare congenital cardiac anomaly. ALCAPA is widely referred to as Bland-White-Garland (BWG) syndrome, and it is estimated to occur in 1/300**,**000 live births and comprise between 0.24 and 0.46% of all cases of congenital heart disease [3–5)].

At birth, infants with ALCAPA are usually asymptomatic because of the presence of physiologic pulmonary hypertension and a patent ductus arteriosus. When pulmonary artery pressure drops a few weeks to months after birth, blood flow reverses from the left coronary artery (LCA) into the pulmonary artery and results in a steal phenomenon. The balance between the speed of closure of the ductus and the development of collateral circulation between the right and left coronary arteries is an important factor in determining the outcome. In the absence of treatment, approximately 90% of infants with ALCAPA die within the first year of life [6)]. Early autopsy studies have reported an average age of sudden death of 35 years old in untreated adult ALCAPA cases, and the authors of those studies have recommended that all diagnosed adult ALCAPA patients be treated surgically [7–9)]. However, little is known about the pathological changes and clinical course of ALCAPA in adults or the association between adult ALCAPA and sudden death.

We were involved in the assessment, treatment, and pathological evaluation of two adult cases of ALCAPA, and we aimed to extend our knowledge of the histological, angiographic, and clinical characteristics of ALCAPA in adults and to achieve a deeper understanding of this rare entity.

## Case presentations

### Case 1

**Fig. 1:**
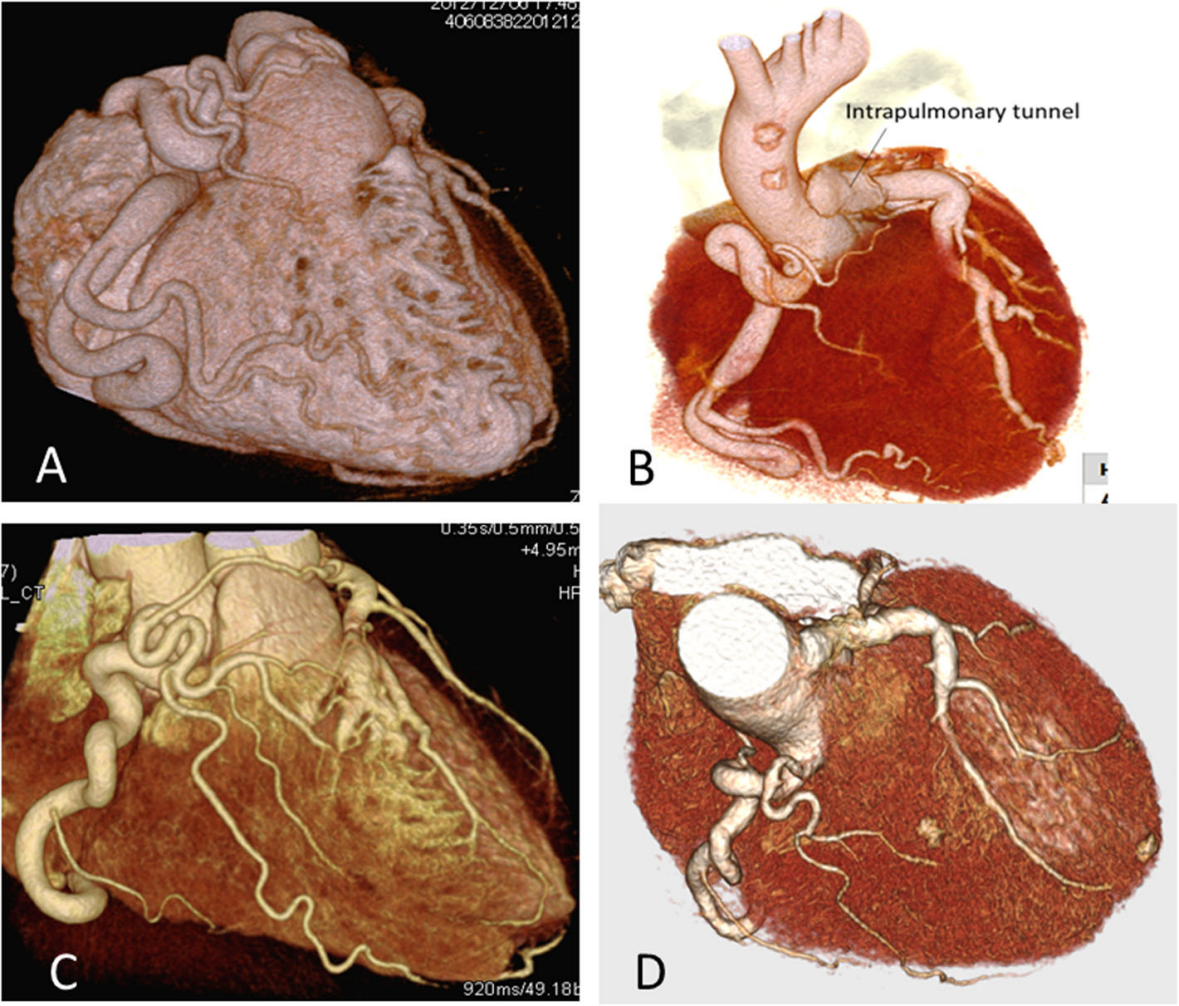
Pre- and post-operative 3D-computed tomography**. a**. Preoperative 3-D CT in Case 1. **b**. Postoperative 3-D CT in Case 1. **c**. Preoperative 3-D CT in Case 2. **d**. Postoperative 3-D CT in Case 2. Preoperative 3-D CT revealed a large RCA with extensive collateral filling of the left coronary vascular territory, and the left main trunk connected directly to the pulmonary trunk. Postoperative 3D-CT confirmed diminished size of the collateral arteries and an anatomically corrected LCA by means of an intrapulmonary baffle tunnel

The patient was a 42-year-old male with a family history of sudden early postnatal death of an elder sister whose cause of death was unknown. The patient pursued an active life without any symptoms of heart failure or arrhythmia before the sudden onset of ventricular fibrillation at 42 years of age. He had collapsed unconscious while jogging, and cardiopulmonary resuscitation (CPR) was performed by a bystander. The ambulance crew found that the patient was in ventricular fibrillation, and they defibrillated him with an automated external defibrillator (AED). Upon arrival at the hospital, he was intubated and placed under mild induced hypothermia at 34°C for 5 days. The patient gradually recovered consciousness, and was extubated. The CAG findings led to a diagnosis of adult ALCAPA (Fig. 1a, b). A modified-Takeuchi procedure was performed, and an ICD was implanted. The patient’s postoperative course was uneventful, and as of 11.5 years after the surgical correction, he is alive and well with no complications and no ICD activations [10)].

### Case 2

The patient was a 48-year-old male with no family history of sudden death or cardiac disease who had been told he had a systolic heart murmur since he was a child. At 42 years of age he experienced a brain infarction and developed dysarthria and left hemiplegia. An electrocardiogram after the brain infarction revealed atrial fibrillation without ischemic change. Coumadin administration was commenced, and he recovered well without sequelae and was followed up at a nearby hospital until the onset of ventricular fibrillation. Ultrasound cardiogram (UCG) 2 months before the onset of ventricular fibrillation showed an enlarged left ventricle (left ventricular diastolic diameter/systolic diameter: 56.4 mm/42.3 mm) and a 50.9% ejection fraction without asynergy. There was moderate mitral valve regurgitation and mild tricuspid regurgitation. Estimated right ventricular systolic pressure was 32.9 mmHg.

At 48 years of age, the patient was found unconscious in bed by his family after a quarrel. Application of an AED by a rescue team restored his heart beat. Upon arrival at the hospital, he was placed under mild hypothermia at 34 °C for 5 days. The patient recovered consciousness without neurological deficits, and was extubated. A modified-Takeuchi procedure, mitral valvuloplasty, and maze procedure were performed (Fig. 1c, d), and were followed by implantation of an ICD. The patient was discharged from the hospital, and he returned to work. However, 9 months after being discharged he was readmitted because of mild symptoms of heart failure: facial edema and weight gain. Ultrasound cardiography at the time showed a left-to-right shunt due to baffle leakage, moderate tricuspid and mitral valve regurgitation, and pulmonary stenosis. The left ventricular diastolic diameter/systolic diameter were 52.0/27.0 mm, and the ejection fraction was 58%.

Swan-Ganz catheterization revealed oxygen step**-**up from 58 to 67% in the pulmonary artery, a mean pulmonary capillary wedge pressure of 27 mmHg, right ventricular systolic/end-diastolic pressure were 79/17 mmHg, and main pulmonary artery pressure was 55/19 (mean: 30) mmHg.

Mitral valve replacement, patch enlargement of the main pulmonary artery, closure of the baffle leakage, and tricuspid annuloplasty were performed 9 months after the initial operation. Oliguria and hyperbilirubinemia developed postoperatively, and the pulmonary hypertension (50–60/ mmHg) persisted. Two months later, a deep sternal surgical wound infection was detected, and sternal debridement was performed. The causative microorganism was methicillin resistant *Staphylococcus aureus*. Four months later, UCG revealed multiple vegetations on the ICD lead and tricuspid ring, and the ICD generator, lead, and tricuspid ring were removed urgently. Six months postoperatively, the patient developed multiple organ failure, and after subsequently developing non-occlusive mesenteric ischemia, he died 22 months after the initial operation.

Three-dimensional computed tomography (3D-CT), coronary angiography (CAG), and gadolinium-enhanced magnetic resonance imaging (MRI) were performed pre- and post-operatively in both patients. Coronary artery blood flow velocity was estimated by CAG, and opacification rates (calculated by averaging the beats required to opacify the vessel) were measured and compared. To perform the histological studies, after obtaining written consent from both patients, an intraoperative myocardial needle biopsy was performed on both the right ventricle (RV) and the left ventricle (LV).

In Case 2, a biopsy at the same sites was performed at the re-do operation to compare the histological findings after coronary artery reconstruction. The RV biopsies were performed transmurally, and the LV biopsies were performed adjacent to the anterior papillary muscle on the endocardial side. Specimens were stained with Elastica van Gieson stain. Samples from other biopsy specimens collected from both ventricles were fixed in 3% glutaraldehyde buffered to pH 7.4 with 0.09 mol/liter of potassium oxalate for use in an electron-microscopic examination.

### Data analysis

All analyses were performed using Excel software (Microsoft, Redmond, Washington, USA). Normally distributed data are presented as the mean + SD.

## Results

### 3D-CT, CAG, and MRI

In both cases, preoperative 3D-CT revealed a large right coronary artery (RCA). Extensive collateral arteries filling the LCA, which connected directly to the pulmonary trunk**,** were present across the free wall of the right ventricle (RV). Postoperative 3D-CT confirmed diminished size of the collateral arteries, an anatomically corrected LCA, and a patent intrapulmonary baffle tunnel (Fig. 1a, b, c, d).

Preoperative CAG revealed a large RCA and extensive collaterals from the RCA to the LCA system. The preoperative RCA opacification rate was 2.35 + 0.08 (average of 2 angiographic angles: *n* = 2) in Case 1 (Additional file 1: Video S1), and 3.32 + 0.65 (*n* = 3) in Case 2. The postoperative opacification rate was 7.36 + 1.12 (*n* = 2) in the RCA, 3.81 + 0.51 (*n* = 3) in the left anterior descending (LAD) artery, and 4.08 + 0.27 (*n* = 4) in the left circumflex (LCx) artery in Case 1 (Additional file 2: Video S2 A, B), and 9.43 + 0.57 (*n* = 3) in the RCA, 3.91 + 0.32 (*n* = 3) in the LAD, and 3.29 + 0.28 (*n* = 2) in the LCx in Case 2.


**Additional file 1**: **Video S1**. Preoperative CAG of Case 1. Preoperative CAG revealed a large RCA and extensive collaterals from the RCA to the LCA system. CAG also showed a “slow flow phenomenon.” The preoperative RCA opacification rate (beats required to opacify the vessel) was 2.35 + 0.08, and it was slower than the normal opacification rates of 1.2–1.3. (MP4 4693 kb)


**Fig. 2:**
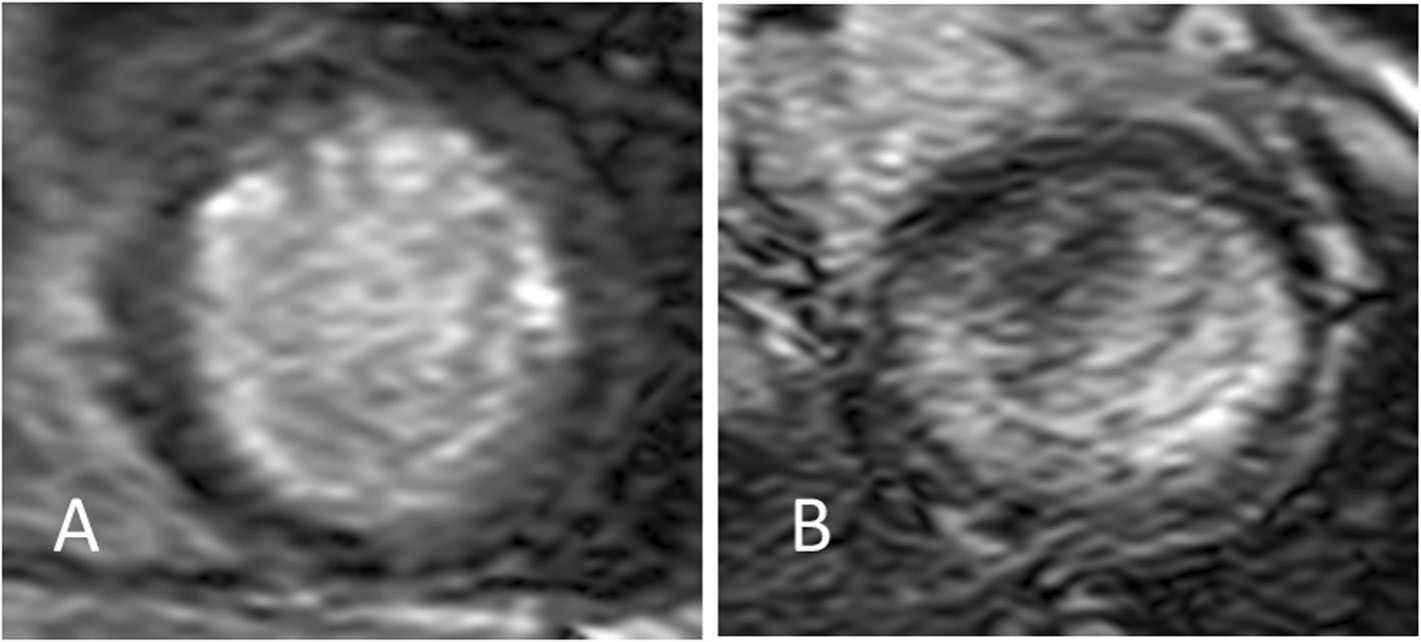
Late gadolinium-enhanced MRI. **a**. In Case 1, subendocardial lesions were detected in the anterolateral area. **b**. In Case 2, a subendocardial laminar lesion was detected in the lateral area

Late gadolinium-enhanced MRI detected subendocardial scar lesions in the anterolateral area in Case 1 (Fig. 2a), and subendocardial scar lesions in the lateral area in Case 2 (Fig. 2b).

### Histological study

**Fig. 3:**
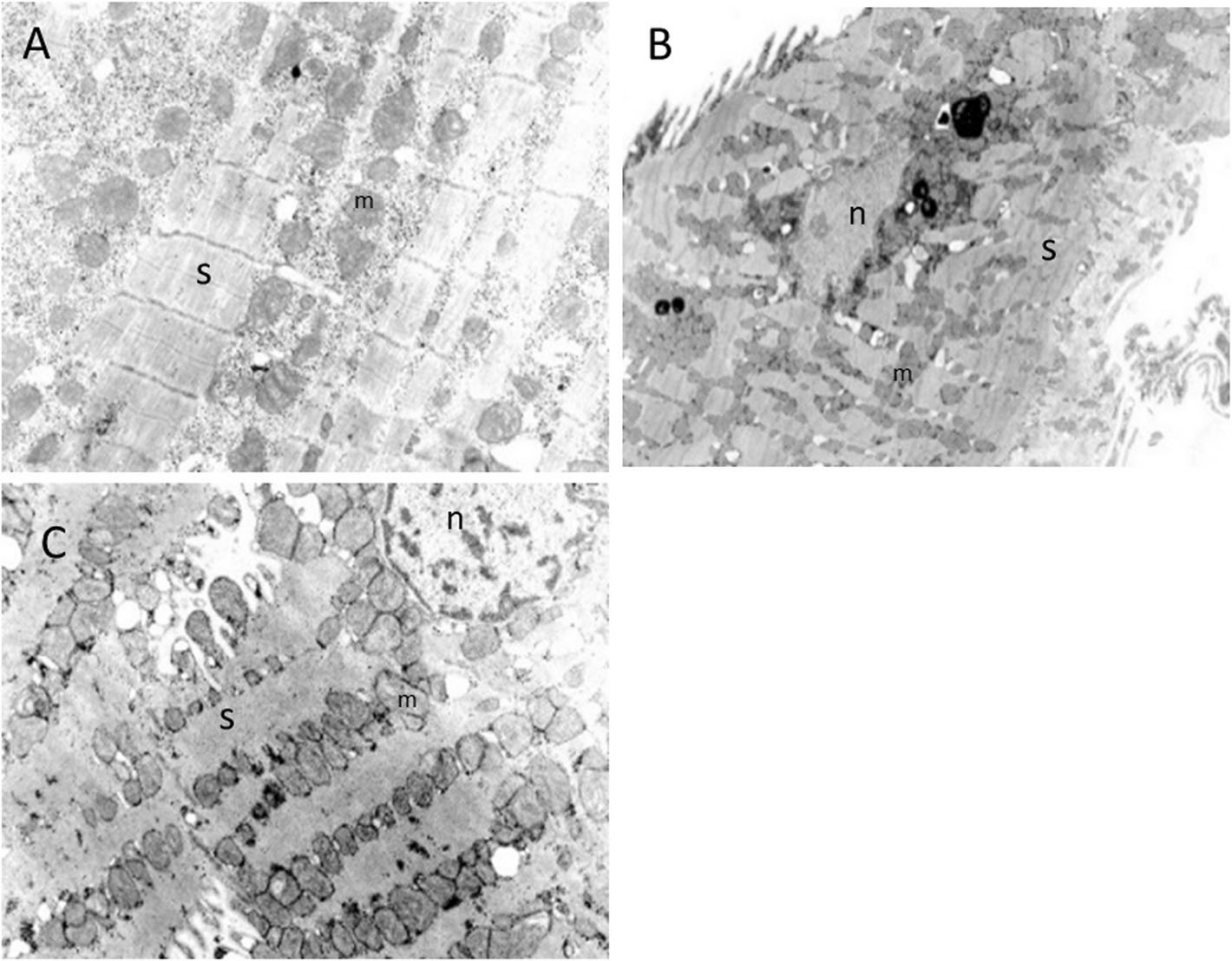
Electron-microscopic findings in the left ventricular myocardium in Case 1. **a**. Ultrastructural examination of the altered myocytes revealed a large number of glycogen granules in the cytoplasm, numerous small mitochondria (m), and dehiscence and a severely reduced volume fraction of sarcomeres (s). **b**. Increased lipofuscin granules and tortuosity of the nuclear membrane (n) are seen. **c**. Mitochondria of various sizes are seen. Strands of distorted sarcomeres without dehiscence are visible. The nuclear membrane has a smooth surface. × 15,000

The electron**-**microscopic examination revealed that some myocytes contained a substantial myofibril fraction, tightly packed rows of mitochondria, and a tortuous nuclear membrane. Some cells showed loss of myofibrils, small rounded mitochondria, giant mitochondria with concentrically arranged cristae, a large accumulation of glycogen, loss of sarcoplasmic reticulum, and nuclear membrane tortuosity. The myocardium was concluded to be viable despite these alterations, because it did not exhibit any degenerative characteristics, such as sarcomere disruption, cytoplasmic vacuoles, cytosolic edema, mitochondrial swelling, cristae, membrane disruption, or accumulation of secondary lysosomes (Fig. 3a, b, c).

**Fig. 4:**
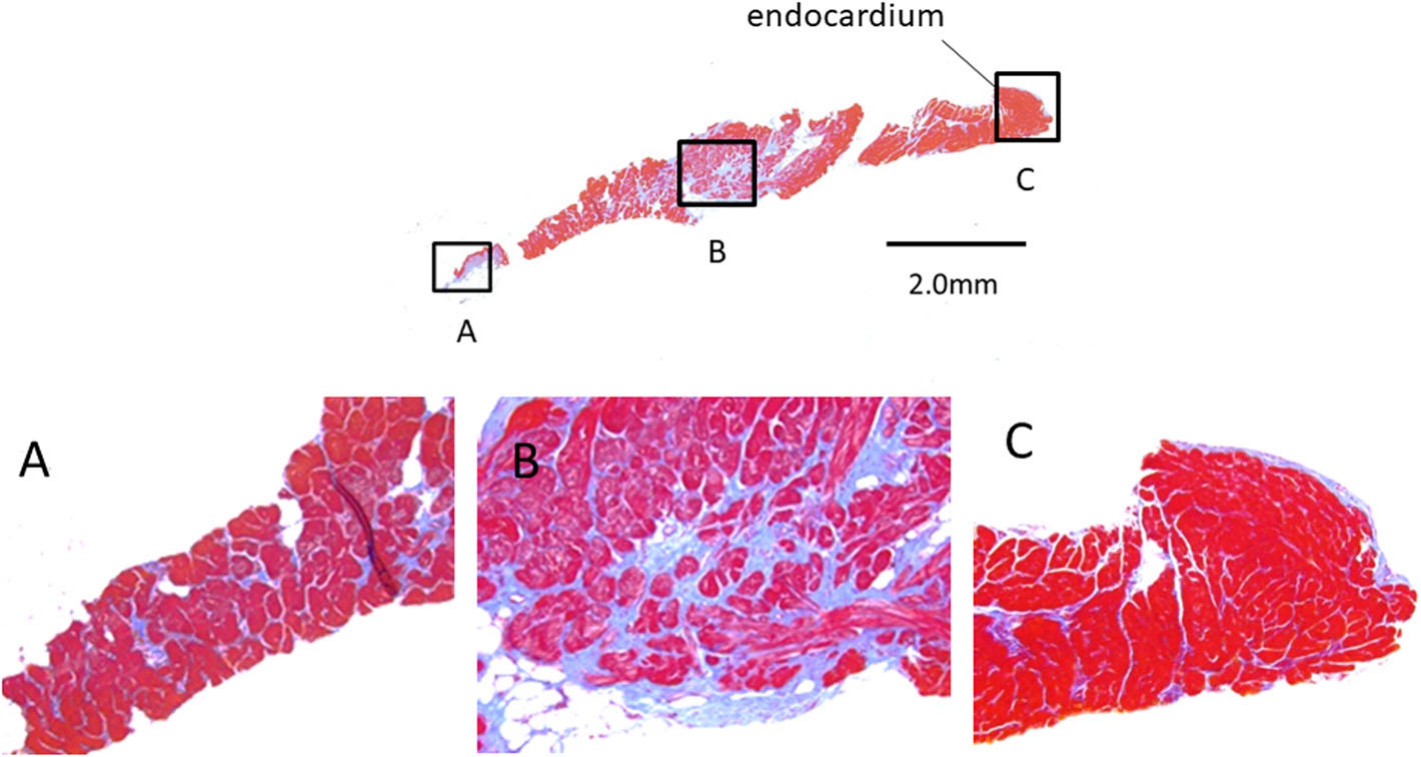
Light-microscopic findings in the right ventricular myocardium in Case 2 at 9 months after the surgical correction. Fibrosis is visible, but it is milder than in the LV. **a**. Epicardial portion of the RV. The myocardium is hypertrophic and exhibits anisokaryosis. **b**. Middle portion of the RV. Fibrosis is visible, but it is milder than in the LV. **c**. Endocardial portion of the RV. The myocardium is hypertrophic

Light microscopy revealed areas of various degrees of altered myocardial structure in both patients. The intraoperative specimens obtained in Case 2 at 9 months after surgical correction continued to show the same ischemic changes, and there was no histological evidence of a reversal of the previous abnormal findings. The RV showed milder structural changes in comparison with the LV (Fig. 4a, b, c).

**Fig. 5:**
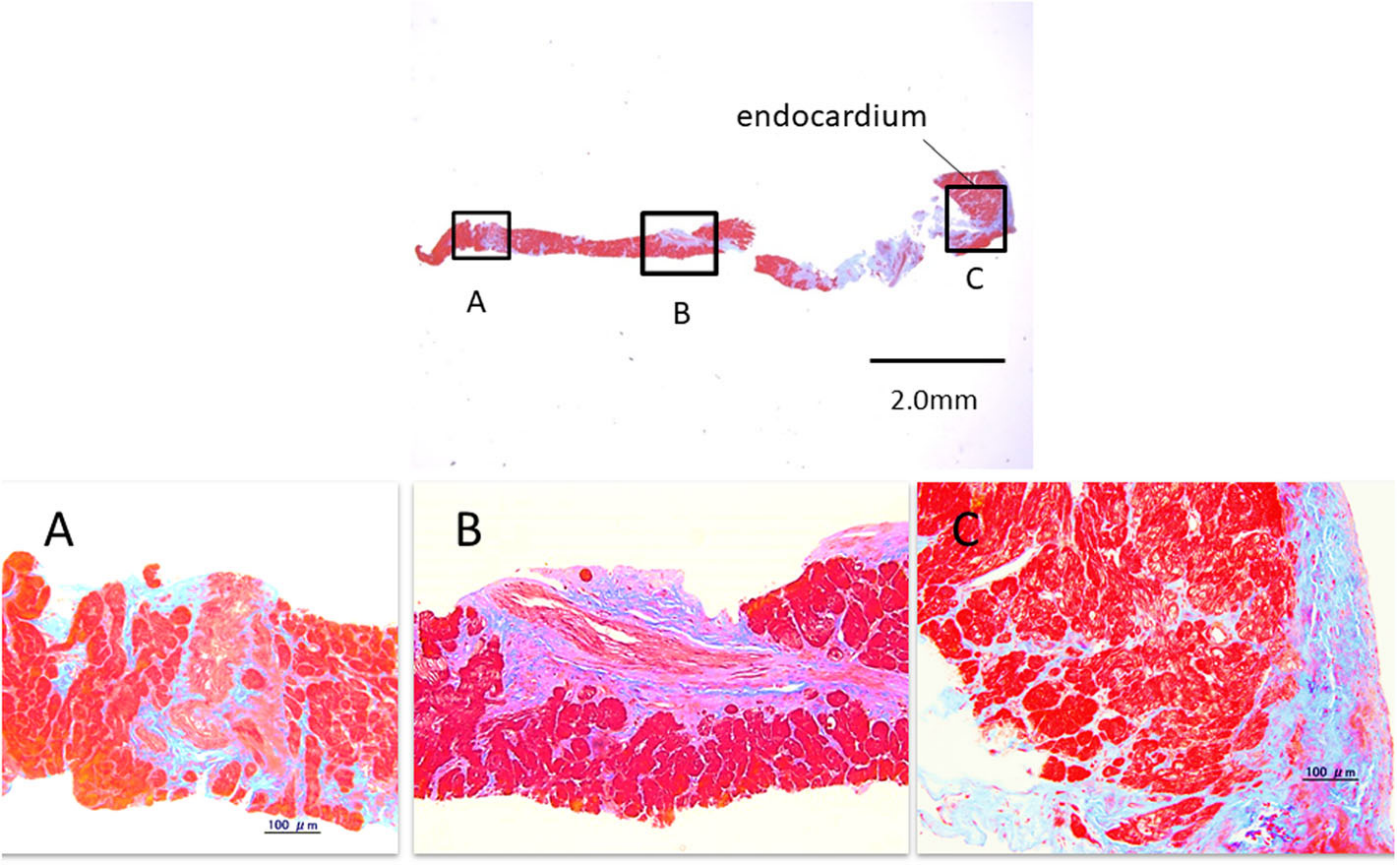
Light**-**microscopic findings in the left ventricular myocardium in Case 2 at 9 months after surgical correction. The myocardium is hypertrophic and exhibits anisokaryosis. Hyalinization and fibrosis of the perivascular stromal tissue, which is compatible with “patchy infarction,” are also visible. **a**. Epicardial portion of the LV. A severely thickened arteriolar wall is visible. The diameter of the arteriolar lumen is significantly decreased. Hyalinization and fibrosis of the perivascular stromal tissue are also visible**. b**. Middle portion of the LV. A severely thickened arteriolar wall is visible. Hyalinization and fibrosis of the perivascular stromal tissue are also visible. **c**. Endocardial portion of the LV. Endocardial fibrosis and hyalinization extending into the myocardium are visible

The LV exhibited hyalinosis, calcification, patchy fibrosis, deformity and acidophilic change of the cytoplasm, anisonucleosis, and blurriness of the striated pattern of the myocardium. Severely thickened arteriolar walls were observed across the entire heart wall, from the endocardial side to the epicardial side (Fig. 5a, b, c).

### EPS and ICD implantation

Neither patient was found to have late potentials or T-wave alternans. An ICD pre-implantation EPS was performed in Case 1. Reproducible poly-morphologic ventricular tachycardia, maximum duration 8 s, was induced by three consecutive right ventricular stimulations, and it resolved spontaneously. Ventricular fibrillation was induced by T-wave shock and ceased at a defibrillation threshold of 10 J. There was no increase in ventricular tachycardia inducibility after isoproterenol administration. In the Bruce protocol treadmill test, the patient’s resting heart rate of 75 beats rose to 181 beats per minute, and his resting systolic blood pressure of 124 mmHg increased to 232 mmHg. His endurance time was 8 min and 1 s, and the test was concluded because of leg fatigue and his heart rate having reached the maximum rate that had been set. No chest symptoms developed during the exercise. Only several premature ventricular contractions were detected, and there were no ST-T changes. In Case 2, the ICD was implanted without performing an EPS.

## Discussion and conclusions

ALCAPA is an extremely rare congenital anomaly. ALCAPA patients usually show clinical signs of cardiac failure at approximately 3 months of age, when their pulmonary arterial pressure decreases, and without treatment, approximately 90% of infants die within the first year of life [7)]. Thus, diagnosis in living adults is extremely rare, and most of the information has been derived from case reports of individual patients who have been resuscitated and survived. Yau et al. reviewed 151 adult cases of ALCAPA, 12% of which were diagnosed at autopsy [11)]. The average age of the patients at the time of diagnosis in those 151 cases was 41 years, and the oldest patient was 83 years old. There was a greater than 2:1 predominance of females. At presentation, 66% of the patients exhibited symptoms of angina, dyspnea, palpitations, or fatigue. Ventricular arrhythmia, syncope, or sudden death was exhibited in 17% of the cases as the initial manifestation, and 14% of the patients were asymptomatic; 62% of those who presented with a life-threatening event had no previous symptoms. The average age of the patients who presented with a life-threatening event was 33 years + 14 years.

### Histological picture

There have been few histological studies of the myocardium of ALCAPA patients, especially in adults. Shivalkar et al. demonstrated variable degrees of fibrosis in a histological study of biopsy specimens taken from the region perfused by the anomalous artery [12)]. The ultrastructure of the remaining myocytes showed viable characteristics, but a substantial percentage contained a markedly reduced fraction of contractile material, reflecting delayed subcellular adaptive responses in the chronically hypoperfused myocardium [13)].

The presence of viable myocytes containing some ultrastructural alterations that were detected electron-microscopically might be interpreted as evidence of a structural adaptation to chronic ischemia [12)]. The degree of the abnormalities varied from an almost normal to a severely altered myocardial structure, depending on the site examined.

Light-microscopic examination revealed a thickened microvasculature and variable amount of “patchy” fibrosis in all of the layers of the cardiac wall. Kristensen et al. state that restoration of a dual coronary system prevents further ischemia and arrhythmias of acute ischemic origin and that the anatomical substrate for ventricular arrhythmias in patients with an old myocardial infarction is unaltered after revascularization [13)]. However, as far as we were able to determine in a review of the literature, no pathological examinations after corrective surgery of ALCAPA had ever been reported. Hong et al. examined pigs in which LAD stenosis had been created, and reported finding that the small intramyocardial coronary arteries in the hibernating myocardial region distal to a flow-limiting coronary artery stenosis had undergone remodeling, and that there was an increase in arteriolar wall thickness and decrease in lumen diameter [14)]. They also observed interstitial, perivascular, and replacement segmental or patchy fibrosis, and the fibrosis was more prominent in the subendocardial region. These structural changes increase vascular resistance and restrict blood flow to ischemic myocardium, and that may account for the pathophysiologic impairment of coronary blood flow. Reduced shear stress leads to greater production of mitogenic and fibrogenic growth factors, including platelet-derived growth factor and transforming growth factors [14)], and low blood flow or reduced shear stress leads to under-expression of growth inhibitors, such as nitric oxide. This suggests that small vessel disease in the presence of ischemia contributes to a further myocardial blood flow reduction, and thereby causes a severer degree of myocardial fibrosis [14)]. Although the ischemic myocardial lesions associated with severe coronary atherosclerosis are generally more prominent in the subendocardial region [15, 16)], our cases showed chronic ischemic change not only in the subendocardial region but uniformly throughout all layers of the heart wall. Since, unlike the ischemia caused by epicardial coronary artery stenosis, the etiology of the ischemia in adult ALCAPA is a steal phenomenon, this uniform pathological ischemic change throughout all of the layers may be a “specific” characteristic of adult ALCAPA.

Cardiac ischemia is often followed by a prolonged decrease in coronary microvascular perfusion, the so-called “no-reflow” phenomenon, even after flow in an upstream artery has been restored, and the mechanism responsible for this phenomenon involves microvascular obstruction [17, 18)]. Myocardial fibrosis is irreversible, even after restoring blood flow, but there has been little investigation of the reversibility of the thickened arteriolar walls. Hong et al. described the increased arteriolar wall thickness in an animal model of myocardial ischemia is caused by intimal hyperplasia with a modest increase of amorphous matrix of the wall. In their study, proliferating cells in the thickened wall stained positive with an α-actin antibody, indicating proliferation of vascular smooth muscle cells [14)]. The increased amorphous matrix and proliferated smooth muscle cells may not have been reversible even after the corrective surgery in adult ALCAPA. Since it is impossible to draw any conclusions based on only one case with a complicated clinical course, further study will be necessary.

### Clinical features

Even before surgery both patients in the cases reported here had an opacification rate that was twice as long as the normal coronary artery opacification rates of 1.2–1.3, i.e., they exhibited a so-called “slow flow phenomenon” [19)]. Increasing vascular resistance due to arteriolar wall thickening is thought to be the main reason for the preoperative prolongation of the RCA opacification rate. Postoperatively, the RCA opacification rate was three times the normal rate, and this further prolongation may have mainly been attributable to the decreased blood flow to the RCA due to the restored antegrade flow in the LCA. The postoperative opacification rate of the RCA was greater than in the LCA*.* In view of the unaltered histological changes, this finding may mainly have been due to the difference in size between the two coronary arteries.

Based on the late gadolinium-enhanced MRI findings, Schmitt et al. documented myocardial scarring caused by ischemia in 65% of adult ALCAPA patients who had undergone surgical correction, although all displayed good LV recovery [20)]. Browne et al. reported the cases of two children with ALCAPA who underwent orthotopic cardiac transplantation, and pathological examination of the cardiac explants in both cases showed extensive fibrotic tissue that correlated with the areas of abnormal delayed enhancement on their MRI scans [21)].

Establishment of a dual coronary system is currently the accepted norm for correction of an ALCAPA in adults. The return of antegrade flow in the LCA has been associated with a reduction in size of the previously dilated RCA and regression of the intracoronary collateral network. Establishment of a dual coronary system can be achieved by various procedures, including a saphenous vein graft, left internal mammary graft, Takeuchi procedure, and direct implantation [22–26)]. Direct implantation is considered technically more difficult and hazardous in adults, but it provides a more physiologic correction and reestablishment of a dual coronary system with a better outcome [27)]. Schwartz et al. reported finding that the degree of preoperative mitral regurgitation is predictive of outcome, but that the severity of preoperative cardiac dysfunction and the magnitude of ventricular dilation are not [28)]. After undergoing an intrapulmonary baffling procedure, the majority of the patients develop supravalvular pulmonary stenosis**,** and several develop baffle leaks; many patients require reoperation for these complications [29)].

Implantation of an ICD in adult ALCAPA patients remains controversial, but it is usually performed because of the presence of such sudden cardiac death risk factors after surgical repair as: (i) severe left ventricular dysfunction, (ii) fibrotic changes as a possible substrate for ventricular arrhythmias, (iii) non-sustained ventricular tachycardia during exercise testing or Holter monitoring, and (iv) a positive programmed ventricular stimulation test [30)].

To date, there has been insufficient data to conclude that surgical repair reduces the risk of recurrent malignant ventricular arrhythmia. Three mechanisms for the development of ventricular tachycardia may be possible: i) acute local ischemia caused by coronary steal phenomena, ii) a reentry circuit in the border zone of myocardial infarction, and iii) electrical instability caused by endocardial fibrosis [30)]. We think that the indications for ICD implantation should be considered more cautiously, because acute ischemic events can be prevented by surgical correction and because there have been no reports of ICD activation after surgical correction, including in our Case 1, and the ICD lead may increase the risk of infection.

Over the past two decades, the number of ALCAPA cases reported in patients over 50 years of age has increased in tandem with the introduction of new noninvasive diagnostic modalities [14)]. Although the natural course of ALCAPA results in a high incidence of sudden death during childhood and early adulthood, the risk of sudden death appears to decline after 50 years of age despite the less common surgical correction in this population, and these older patients, in whom cardiac surgery is undesirable or unwarranted, continue to be diagnosed at an increasing rate [11)]. Special attention should be paid to preventing lethal events in this untreated population. Although no standard protocol of medical control has yet been established, medical control with a vasodilator and anti-arrhythmic agent may be mandatory [31)].

In summary, we have confirmed the previously seldom reported i) electron-microscopic findings and ii) light-microscopic findings in adult cases of ALCAPA, and we have described new findings: iii) arteriolar wall thickening, iv) persistent altered ultrastructure even after surgical correction, and v) a slow flow phenomenon in the CAG in adult ALCAPA.

Because no randomized controlled trials that would make it possible to evaluate the long-term outcomes of methods of managing adult ALCAPA are yet available, compiling systematic descriptions of the data in each case would reveal the true nature of this rare entity and its optimal treatment.

## Supplementary information

**Additional file 2: Video S2.** Postoperative CAG in Case 1. -A. Postoperative CAG showed a well-established dual coronary system. The RCA showed a severer “slow flow phenomenon.” The opacification rate was 7.36 + 1.12. -B. The LCA showed a “slow flow phenomenon”. The opacification rate was 3.81 + 0.51 in the LAD and 4.08 + 0.27 in the LCx. These rates are markedly slower than the normal opacification rates of 1.2–1.3.

## Data Availability

Supporting data are available upon request to the corresponding author. Publication of raw data is impossible as it would conflict with our privacy policy.

## Abbreviations

3D-CT: three-dimensional computed tomography
AED: Automated external defibrillator
ALCAPA: Anomalous left coronary artery from the pulmonary artery
CAG: Coronary angiography
CPR: Cardiopulmonary resuscitation
ECG: Electrocardiogram
EPS: Electrophysiological study
ICD: Implantable cardioverter defibrillator
LAD: Left anterior descending
LCA: Left coronary artery
LCx: Left circumflex
LV: Left ventricle
MRI: Magnetic resonance imaging
RCA: Right coronary artery
RV: Right ventricle
UCG: Ultrasound cardiogram
